# Venous Thromboembolism and Bleeding with Temozolomide-, Bevacizumab-, and Nitrosourea-Based Therapy in Glioma: A Dual-Database Pharmacovigilance Study

**DOI:** 10.3390/cancers18010130

**Published:** 2025-12-30

**Authors:** Xiaohong Hou, Yuanlu Zhang, Cheng Liang, Shengtao Yao

**Affiliations:** Department of Neurosurgery, Affiliated Hospital of Zunyi Medical University, Zunyi 563000, China; m13048503352@163.com (X.H.); 15585220267@163.com (Y.Z.); 13116347670@163.com (C.L.)

**Keywords:** malignant glioma, bevacizumab, temozolomide, pharmacovigilance, spontaneous adverse event reporting

## Abstract

**Introduction**: Patients with malignant glioma are inherently at high risk of venous thromboembolism (VTE) and bleeding, and systemic therapy may further modify this vascular risk. This study used large spontaneous reporting databases to compare VTE, central nervous system (CNS) bleeding and gastrointestinal (GI) bleeding signals associated with four representative systemic therapies for glioma—temozolomide, bevacizumab and the nitrosourea alkylating agents lomustine and carmustine—and explore bevacizumab-based combination regimens. **Methods**: We conducted a retrospective pharmacovigilance study using the FAERS and the CVARD, identifying reports of patients with gliomas exposed to temozolomide, bevacizumab, lomustine or carmustine. VTE, CNS bleeding and GI bleeding were defined a priori, and disproportionality was assessed using reporting odds ratios (RORs) with 95% confidence intervals (CIs); a positive signal was defined as a ≥ 3 and a 95% CI lower bound > 1. In bevacizumab-exposed patients, we compared bevacizumab monotherapy with bevacizumab + temozolomide and bevacizumab + lomustine treatments and performed sensitivity analyses on a stricter glioma cohort. **Results**: Bevacizumab was the only drug that consistently showed positive signals for VTE and GI bleeding, whereas temozolomide showed no clear VTE signal, and nitrosourea agents yielded only sparse, unstable increases. Bevacizumab + temozolomide regimens had higher RORs for VTE and GI bleeding than bevacizumab alone, while bevacizumab + lomustine showed only modest VTE increases and no robust bleeding signals. **Conclusions**: In real-world pharmacovigilance data, bevacizumab-containing therapy, particularly when combined with temozolomide, carries the greatest disproportionate burden of VTE and GI bleeding among common systemic treatments for malignant glioma, whereas temozolomide alone appears largely neutral, and current evidence supporting nitrosourea-related signals remains inconclusive.

## Simple Summary

Malignant gliomas are highly aggressive brain tumors, and many patients receive long-term chemotherapy treatment with systemic agents such as temozolomide, bevacizumab and nitrosourea. These patients are already at high baseline risk of venous thromboembolism (VTE) and intracranial or gastrointestinal bleeding because of the tumor itself, surgery, immobility and supportive care. However, it is unclear how much additional risk is contributed by individual drugs and specific treatment regimens in real-world practice. In this study, we analyzed two large drug safety databases, the FAERS and the CVARD, to explore signals of VTE and bleeding associated with these key glioma therapies. We found that bevacizumab-containing regimens showed the clearest and most consistent signals for both VTE and major bleeding, whereas temozolomide monotherapy did not show a stable increase in signal, and nitrosourea-based therapy had weaker or inconsistent signals. Combination analyses suggested that bevacizumab plus temozolomide may carry a higher vascular risk than bevacizumab alone. These findings cannot replace controlled clinical trials, but they provide clinicians with complementary real-world evidence to help balance the benefits of tumor control against the potential for thrombotic and bleeding complications when individualizing treatment for patients with malignant glioma.

## 1. Introduction

Malignant gliomas, particularly glioblastoma, are among the most common and most lethal primary malignant tumors of the adult central nervous system, characterized by high recurrence and mortality rates [1,2]. Despite advances in surgery, radiotherapy and systemic therapy, median survival remains limited, and many patients experience early disease progression [3]. The tumor itself can substantially increase the risk of venous thromboembolism (VTE) through multiple mechanisms, including tumor-associated hypercoagulability, prolonged immobility, surgical interventions and long-term corticosteroid use [4,5]. Reported VTE rates in glioma often exceed those observed in many other solid tumors, and thrombotic events are a major cause of morbidity and mortality [6,7]. Concurrently, intracranial tumor infiltration, vascular abnormalities and treatment-induced thrombocytopenia and mucosal injury predispose patients to bleeding events—especially central nervous system (CNS) and gastrointestinal (GI) bleeding—which are common and serious complications in this population [8,9]. Thrombotic and bleeding events not only directly contribute to mortality and disability but also limit the dosing and continuity of antitumor therapy, thereby further exacerbating the overall clinical burden [10]. The systematic assessment of thrombotic and bleeding safety in the context of systemic therapy for malignant glioma is, therefore, of considerable clinical importance.

Current systemic treatment of glioma is largely based on combined chemoradiotherapy [11]. The oral alkylating agent temozolomide has become the backbone of first-line standard regimens for high-grade glioma and is widely used in both concurrent and adjuvant phases of radiotherapy [12]. Traditional nitrosourea-class agents, such as lomustine and carmustine, have long been used as chemotherapies for recurrent or refractory disease or as components of salvage regimens [13,14]. With the advent of targeted therapy, the anti-VEGF monoclonal antibody bevacizumab has gained widespread use in patients with recurrent glioblastoma and related entities [15]. Multiple randomized controlled trials and meta-analyses have shown that while bevacizumab can improve or alleviate symptoms, it increases the risk of arterial and venous thromboembolism and bleeding; this is biologically plausible because VEGF-A inhibition by bevacizumab can induce endothelial dysfunction, impair vascular repair and reduce vasodilatory and antithrombotic mediators such as nitric oxide and prostacyclin, thereby promoting both thrombotic and bleeding events under conditions of fragile tumor vasculature [16,17]. In clinical practice, it is often combined with temozolomide or nitrosoureas, further complicating the overall thrombotic and hemorrhagic risk profile at the regimen level. However, in the specific context of glioma, existing evidence regarding VTE and bleeding risk with these agents largely comes from clinical trials or single-center cohorts with limited sample sizes, restrictive eligibility criteria and substantial heterogeneity in treatment patterns [18]. These studies do not fully capture the impact of different drugs and commonly used combination regimens on the thrombotic and bleeding risk spectrum in real-world practice. In particular, comparative evaluations of VTE and bleeding adverse reactions among temozolomide-based standard chemoradiotherapy, traditional nitrosourea therapy and bevacizumab-containing regimens in glioma populations remain scarce.

Spontaneous adverse event reporting databases are essential tools for post-marketing safety evaluation. In the absence of large prospective studies dedicated to glioma, they can provide early “signals” of potential drug–event associations and help generate hypotheses on rare but clinically important complications. The FDA Adverse Event Reporting System (FAERS) and the Canada Vigilance Adverse Reaction Database (CVARD) are large real-world data sources representing different health care systems and regulatory environments [19,20]. Disproportionality measures based on 2 × 2 contingency tables—such as the reporting odds ratio (ROR) and proportional reporting ratio (PRR)—as well as Bayesian shrinkage metrics (e.g., information component and its lower-bound IC_025_, and empirical Bayes geometric mean and its lower-bound EB_05_), have been widely used for signal detection in spontaneous reporting systems. These approaches enable a comparative assessment of reporting patterns for specific drug–event pairs across drugs and databases, even when precise exposure denominators are not available. To date, however, no systematic study has focused specifically on glioma systemic therapies and integrated multiple databases to compare disproportionality for serious outcomes such as VTE, CNS bleeding and GI bleeding while also exploring potential safety differences among bevacizumab-based combination regimens.

Against this background, we used the FAERS and the CVARD to assemble a cohort of patients with glioma receiving systemic therapy and prespecified four clinically representative agents—temozolomide, bevacizumab, lomustine and carmustine—as the drugs of interest. Using ROR as the primary disproportionality metric and PRR, IC (IC_025_) and EBGM (EB_05_) as secondary methodological references, we systematically evaluated the disproportionality patterns linking these drugs with VTE, CNS bleeding and GI bleeding across both databases. We further compared thrombotic and bleeding signals for bevacizumab monotherapy versus bevacizumab + temozolomide and bevacizumab + lomustine regimens and tested the robustness of the key findings in a sensitivity analysis based on a stricter glioma case definition. We aimed to delineate the thrombotic and bleeding risk spectrum of systemic therapies for malignant glioma and provide pharmacovigilance evidence that may inform individualized risk assessment, the optimization of monitoring strategies and the design of future prospective studies.

## 2. Methods

### 2.1. Study Design and Data Sources

**Figure 1:**
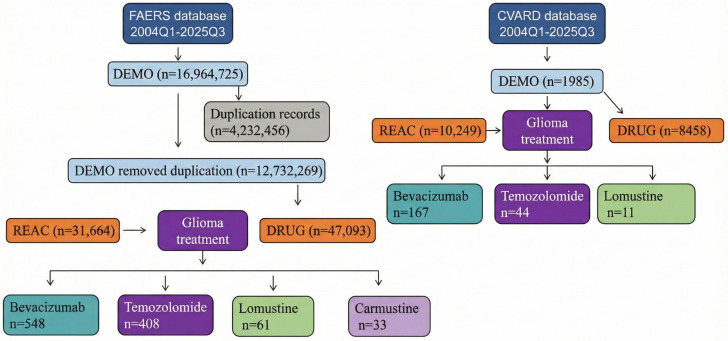
Flowchart for screening adverse events of bevacizumab, temozolomide, lomustine and carmustine from the FAERS and the CVARD.

This was a retrospective pharmacovigilance study based on two spontaneous reporting systems: the FDA Adverse Event Reporting System (FAERS) and the Canada Vigilance Adverse Reaction Database (CVARD). All individual case safety reports (ICSRs) recorded in the two databases from January 2004 to March 2025 were included in the initial dataset. Following official deduplication procedures for each database, for cases with multiple versions, the latter were merged, and only the latest version was retained for analysis (Figure 1). All adverse events were coded using the Medical Dictionary for Regulatory Activities (MedDRA), and the analyses were conducted at the preferred term (PT) level.

### 2.2. Identification of Glioma Cases and Study Population

To construct the study cohort, we searched the indication and medical history/diagnosis fields in the FAERS and the CVARD by using case-insensitive free-text matching to identify reports suggestive of glioma. Any report containing the terms “GLIOMA,” “MALIGNANT GLIOMA,” “GLIOBLASTOMA,” “GLIOBLASTOMA MULTIFORME,” “ASTROCYTOMA,” “ANAPLASTIC ASTROCYTOMA,” “OLIGODENDROGLIOMA,” and “ANAPLASTIC OLIGODENDROGLIOMA” in any relevant field was included in the “glioma cohort.” This relatively broad case definition was chosen to maximize sensitivity for capturing malignant glioma, but it may have also resulted in some misclassification, for example, the inclusion of lower-grade gliomas or non-malignant CNS tumors labeled simply “glioma,” “astrocytoma” or “oligodendroglioma” and the exclusion of some true malignant glioma cases coded under other CNS neoplasm terms. Such misclassification is expected to be largely non-differential with respect to the individual study drugs and would thus tend to dilute contrasts in disproportionality (biasing RORs toward the null) rather than to create spurious strong signals. To partly address this concern, we performed a prespecified sensitivity analysis using a stricter malignant glioma definition (retaining only “malignant glioma,” “glioblastoma,” “glioblastoma multiforme” and “anaplastic astrocytoma”) and re-estimated bevacizumab-related disproportionality for VTE and bleeding. These search terms were specified in English and applied in a case-insensitive manner; in both the FAERS and the CVARD, the indication fields in oncology reports are predominantly recorded using standard English tumor names aligned with MedDRA terminology, so any loss of cases due to non-English diagnostic phrases is expected to be limited. Because information on tumor grade and molecular markers (e.g., IDH mutation and 1p/19q codeletion) is limited in spontaneous reporting systems and because the recording of tumor diagnoses is heterogeneous, we did not further stratify cases by WHO grade or molecular subtype. In the main analysis, we did not subdivide cases by specific pathological subtype (e.g., glioblastoma versus other high-grade gliomas). The included reports were considered to represent a population of patients with malignant glioma/glioblastoma receiving systemic therapy.

### 2.3. Selection of Study Drugs and Exposure Definition

Given our aim to characterize the thrombotic and bleeding safety profile of systemic therapy in glioma, we prespecified four drugs commonly used in clinical practice as the study agents: the oral alkylating agent temozolomide, the anti-VEGF monoclonal antibody bevacizumab and the nitrosourea-class alkylating agents lomustine and carmustine, which together were considered nitrosourea-based therapy in this study. In the CVARD, however, glioma treatment-related reports involving carmustine were extremely sparse and did not yield analyzable VTE or bleeding events (a < 3), so carmustine could only be evaluated as a separate exposure group in the FAERS. In FAERS and CVARD, carmustine exposure was identified at the generic-drug level (including mapped brand names such as Gliadel), but route of administration and formulation (intravenous infusion vs. intracavitary wafer implantation) are not systematically captured; therefore, the carmustine group likely comprises a mixture of systemic and wafer-based use in contemporary glioma practice. Together, these drugs constitute the core systemic treatment options for high-grade or relapsed glioma, encompassing traditional nitrosourea-based regimens, as well as temozolomide-based standard chemoradiotherapy and bevacizumab-containing modern targeted strategies. In both databases, drug names were standardized at the generic level, with all brand names mapped to their corresponding generic names before aggregation. Any report in which a target drug appeared in any role (primary suspect, secondary suspect or concomitant) was regarded as “exposed,” whereas reports without that drug were classified as “unexposed,” because drug–role coding is heterogeneous in oncology ICSRs and a broader definition reduces the under-ascertainment of true exposure for disproportionality-based signal detection. Information on cranial radiotherapy was not systematically available in structured fields in either the FAERS or the CVARD, and radiotherapy recorded only in free-text narratives could not be consistently extracted; therefore, concomitant radiation therapy was not modeled as separate exposure or a covariate.

### 2.4. Definition of Outcome Events

We focused on three clinically important thrombotic and bleeding outcomes:

Venous thromboembolism (VTE), including PTs for deep vein thrombosis, pulmonary embolism and related venous thrombosis/embolism events.

Central nervous system (CNS) bleeding, including PTs for intracranial hemorrhage, cerebral hemorrhage, intracerebral hematoma and other CNS-related hemorrhagic events.

Gastrointestinal (GI) bleeding, including PTs such as “gastrointestinal hemorrhage,” “upper/lower gastrointestinal hemorrhage,” “hematemesis” and “melaena,” indicating GI bleeding.

For each composite endpoint, any ICSR that included ≥1 of the corresponding MedDRA PTs was counted once for that endpoint; we did not attempt to distinguish or count multiple episodes within the same report. The PT groupings for each endpoint were defined a priori based on clinical relevance and consensus among investigators with expertise in neuro-oncology and clinical pharmacy to ensure both clinical interpretability and methodological feasibility. The FAERS and the CVARD do not consistently distinguish between initial and recurrent events; therefore, each endpoint encompassed any reported VTE or bleeding episode, irrespective of whether it represented a first occurrence or a recurrence.

### 2.5. Disproportionality Measures and Signal Detection

To assess the disproportionality of drug–event pairs in the spontaneous reporting databases, we used the reporting odds ratio (ROR) derived from 2 × 2 contingency tables as the primary effect measure and basis for signal detection. For each drug–event combination in each database, a table was constructed with the following cells: a, “target drug + target event”; b, “target drug + other events”; c, “other drugs + target event”; and d, “other drugs + other events.” The RORs were calculated as (a/c)/(b/d), and their 95% CIs were estimated. A positive signal was defined as a ≥ 3 and a lower bound of the 95% CI for the ROR > 1. These disproportionality metrics were used to identify potential safety signals in terms of disproportionate reporting and were interpreted as hypothesis-generating associations rather than measures of absolute risk or causal effects.

In addition, PRRs with 95% CIs and Bayesian shrinkage metrics, including IC with its 95% credibility lower bound (IC_025_) and EBGM with its 5% lower bound (EB_05_), were calculated as secondary measures and for methodological sensitivity analyses. PRRs and their 95% CIs were used to complement the RORs in describing differences in reporting proportions between drugs. IC/IC_025_ and EBGM/EB_05_ were used to shrink extreme estimates in situations with low counts or sparse distributions and verify the direction and magnitude of ROR-based signals from a Bayesian perspective. To avoid excessively conservative multi-threshold criteria that might obscure true signals, we used RORs and their 95% CI as the primary basis for formal signal detection decisions. PRRs and IC/EBGM were treated as secondary methodological triangulation; when an ROR suggested a stable positive signal but IC_025_ or EB_05_ were more conservative, such findings were interpreted as statistically suggestive but requiring cautious interpretation.

### 2.6. Main Disproportionality Analysis for Four Glioma Therapies

In the main disproportionality analysis, we first identified all ICSRs within the glioma cohort that mentioned temozolomide, bevacizumab, lomustine or carmustine and treated these four agents as prespecified exposure. The FAERS and the CVARD were analyzed independently. For each database, 2 × 2 tables were constructed for each drug–event combination: “target drug + target event” (a), “target drug + other events” (b), “non-target drugs + target event” (c) and “non-target drugs + other events” (d). Patients in any report that included the generic name of interest in any role (primary or secondary suspect drug or concomitant medication) were considered exposed.

The three prespecified endpoints—VTE, CNS bleeding and GI bleeding—were defined as clinically coherent PT groupings based on MedDRA. For example, PTs for deep vein thrombosis and pulmonary embolism were grouped under VTE; intracranial and cerebral hemorrhage PTs under CNS bleeding; and GI hemorrhage, hematemesis and melaena PTs under GI bleeding. RORs with 95% CIs were calculated as the primary disproportionality metrics for each drug–endpoint pair, and combinations meeting the criteria of a ≥ 3 and a 95% CI lower bound > 1 were considered to have positive disproportionality signals. To facilitate cross-drug and cross-database comparison, identical endpoint definitions and signal detection rules were applied in the FAERS and the CVARD, and ROR distributions and signal patterns for the four drugs across the three outcomes were presented side by side to characterize the thrombotic and bleeding signal spectrum associated with glioma systemic therapies.

### 2.7. Bevacizumab Regimen Stratification and Comparisons

For the bevacizumab-based combination subgroup analysis, we first identified all ICSRs in the glioma cohort that mentioned bevacizumab and then categorized them into three mutually exclusive regimen groups according to concomitant chemotherapy: (1) bevacizumab monotherapy (reports containing bevacizumab but neither temozolomide nor lomustine); (2) bevacizumab + temozolomide; and (3) bevacizumab + lomustine. Grouping was performed separately in the FAERS and the CVARD. Bevacizumab combined with carmustine was reported only rarely, and the corresponding numbers of VTE and bleeding events did not meet the prespecified minimum cell count (a < 3) for disproportionality analysis; therefore, this regimen was not evaluated as a separate combination group.

For each bevacizumab-based regimen, 2 × 2 tables were constructed for the three prespecified endpoints (VTE, CNS bleeding and GI bleeding). Reports with “a specific bevacizumab-containing regimen + target event” were counted as cell a, “the same regimen + non-target events” as b, “other bevacizumab-containing regimens + target event” as c and “other bevacizumab-containing regimens + non-target events” as d. RORs with 95% CIs were calculated to evaluate the degree of disproportionality for each bevacizumab-containing regimen and endpoint. For clinical interpretability, bevacizumab monotherapy served as the reference regimen in the presentation and interpretation of results, and particular attention was paid to changes in ROR magnitude and statistical significance for bevacizumab + temozolomide and bevacizumab + lomustine relative to monotherapy across endpoints.

This subgroup analysis was considered exploratory and hypothesis-generating, given the limited clinical detail and potential confounding by indication in spontaneous reporting data.

### 2.8. Sensitivity Analysis Design and Robustness Assessment

Given the potential impact of case definition on the results of spontaneous reporting analyses, we prespecified a disease-definition-based sensitivity analysis to assess the robustness of bevacizumab-related signals. In the main analysis, the glioma cohort was defined by the presence of any of the terms “glioma,” “malignant glioma,” “glioblastoma,” “glioblastoma multiforme,” “astrocytoma,” “anaplastic astrocytoma,” “oligodendroglioma” and “anaplastic oligodendroglioma” in relevant diagnostic fields. In the sensitivity analysis, the cohort was restricted to cases with higher-certainty malignant diagnoses, retaining only reports explicitly labeled as “malignant glioma,” “glioblastoma,” “glioblastoma multiforme” and “anaplastic astrocytoma.” RORs and signal status for bevacizumab-related VTE, CNS bleeding and GI bleeding were recalculated in this restricted cohort and compared with the main analysis. We did not perform separate case-definition sensitivity analyses for temozolomide, lomustine or carmustine because the corresponding VTE/bleeding event counts were relatively small in the main analysis and further restriction was expected to yield unstable estimates.

In addition, to address concerns about exposure misclassification related to drug–role coding, we conducted a bevacizumab-specific sensitivity analysis restricted to primary suspect (PS-only) reports. In this analysis, bevacizumab exposure in the FAERS and the CVARD was limited to ICSRs in which bevacizumab was coded as a primary suspect drug, while the outcome definitions, comparator groups and signal detection rules (RORs and 95% CIs) were kept identical to those in the main analysis. RORs for bevacizumab-related VTE, CNS bleeding and GI bleeding under the PS-only definition were compared qualitatively with those from the main any-role exposure definition.

### 2.9. Statistical Analysis

All data analyses were performed using R software (version 4.4.3).

## 3. Results

### 3.1. Baseline Demographic and Reporting Characteristics

Across the two databases, baseline characteristics of adverse event reports related to glioma therapy showed broadly similar features: a predominance of younger to middle-aged male patients, substantial missing data in certain fields and modest differences among drugs and between databases (Table 1).

In terms of sex distribution, in the FAERS, reports for each drug involved slightly more men than women, and approximately 40% of bevacizumab-related reports lacked sex information, whereas sex data were more complete for temozolomide. In the CVARD, reports involving bevacizumab and temozolomide were predominantly about male patients (about 56–70%), with a lower proportion of missing sex data than in the FAERS.

Most reports in both databases involved patients aged 18–64 years, with a smaller proportion aged ≥65 years. In the FAERS, more than one-third of bevacizumab- and temozolomide-related reports had missing age data, whereas age information was more complete in the CVARD. Body weight was frequently missing in both systems. In the FAERS, among reports with available data, the 50–100 kg category was the most common, but more than 60% of reports lacked weight records. In the CVARD, although a large proportion of reports also had missing or unclassified weight, a non-negligible subset of patients weighed <50 kg, suggesting that malnutrition or cachexia may be prevalent among real-world glioma patients.

Regarding the reporter type, approximately one-half to two-thirds of reports were submitted by health care professionals. In the FAERS, around 60% of bevacizumab- and temozolomide-related reports originated from medical staff, and in the CVARD, more than 70% of lomustine-related reports were submitted by health care professionals. Serious events were not uncommon. In the FAERS, serious event reports (as classified by the database) accounted for approximately 7–23% of drug-specific reports, with lomustine having a slightly higher proportion and carmustine a slightly lower one. In the CVARD, serious outcomes comprised roughly 15–24% of reports involving bevacizumab, temozolomide or lomustine. Overall, serious outcomes were frequent among adverse event reports related to glioma treatment, but the differences among drugs were modest. As these proportions are calculated within spontaneously reported ICSRs, they reflect the enrichment of more severe events in pharmacovigilance systems and cannot be interpreted as incidence rates among all glioma patients treated with temozolomide or other study drugs.

### 3.2. Disproportionality Analysis for VTE and Bleeding Events

In the analysis of VTE, the four systemic therapies for glioma exhibited distinct disproportionality patterns across the FAERS and the CVARD (Table 2). In both databases, bevacizumab consistently showed positive disproportionality signals: the RORs were 2.26 (95% CI: 1.87–2.73) in the FAERS and 1.46 (95% CI: 1.03–2.07) in the CVARD, both meeting the prespecified signal criterion of a ≥ 3 and a 95% CI lower bound > 1. These findings indicate that in real-world glioma treatment, the reporting proportion of VTE following bevacizumab exposure is significantly higher than that associated with other drugs.

By contrast, temozolomide showed RORs of 0.99 (95% CI: 0.82–1.19) in the FAERS and 0.62 (95% CI: 0.39–0.98) in the CVARD, approximating unity in the FAERS and falling below 1 in the CVARD, with no evidence of excess VTE reporting overall. Lomustine demonstrated moderate signal strength in the FAERS (ROR 1.43, 95% CI: 1.06–1.95) but unstable estimates with wide CIs in the CVARD (ROR 1.16, 95% CI: 0.59–2.29). Carmustine did not show robust signals in either database (FAERS: ROR 1.43, 95% CI: 0.88–2.33). Taken together, bevacizumab was the only drug that displayed a stable positive VTE signal in both the FAERS and the CVARD, whereas temozolomide appeared neutral or even mildly protective, and nitrosourea agents showed only limited signal increase in selected analyses.

For bleeding outcomes, the FAERS revealed differentiated signal profiles for the four drugs with respect to CNS and GI bleeding (Table 3a). Bevacizumab was associated with pronounced disproportionality for both endpoints: the RORs were 1.53 (95% CI: 1.23–1.89) for CNS bleeding and 2.30 (95% CI: 1.64–3.21) for GI bleeding, both fulfilling the signal criteria and indicating markedly increased reporting proportions of intracranial and GI hemorrhage among bevacizumab-treated glioma patients. Temozolomide showed an ROR slightly below unity for CNS bleeding (0.81, 95% CI: 0.65–1.00), with no clear disproportionality, and an ROR of 1.40 (95% CI: 1.01–1.94) for GI bleeding. Although the point estimate for GI bleeding modestly exceeded 1, the combination of Bayesian shrinkage measures and prespecified thresholds did not support classification as a robust positive signal, suggesting that the bleeding signal for temozolomide is less prominent than that for bevacizumab. For lomustine, the RORs for CNS and GI bleeding were 0.35 (95% CI: 0.18–0.67) and 0.45 (95% CI: 0.18–1.10), respectively, indicating lower-than-expected or uncertain reporting proportions. Carmustine showed some degree of disproportionality for CNS bleeding (ROR 1.76, 95% CI: 1.04–2.97), whereas GI bleeding reports were too sparse to support firm conclusions.

The results from the CVARD were broadly consistent with those from the FAERS but showed sparser and more asymmetric patterns across specific drug–event combinations (Table 3b). Bevacizumab exhibited a stronger GI bleeding signal in the CVARD (ROR 3.70, 95% CI: 1.44–9.53), while the ROR for CNS bleeding was 1.57 (95% CI: 0.65–3.82); the latter estimate was imprecise, with a wide CI crossing 1, reflecting instability due to limited case numbers. Notably, temozolomide in the CVARD was associated with a more marked GI bleeding signal (ROR 3.33, 95% CI: 1.73–6.42) compared with the FAERS, while still lacking a clear CNS bleeding signal (ROR 0.74, 95% CI: 0.31–1.78). For lomustine, only two GI bleeding reports were identified (a = 2; ROR 1.04, 95% CI: 0.25–4.33), precluding robust inference. Overall, bevacizumab demonstrated highly consistent positive signals across databases for VTE and GI bleeding, whereas a clear CNS bleeding signal was observed only in the FAERS. Temozolomide did not exhibit disproportionate VTE reporting but showed stronger GI bleeding disproportionality in the CVARD than in the FAERS, suggesting that differences in reporting structure and treatment context between databases may influence sensitivity for certain drug–event combinations.

**Figure 2:**
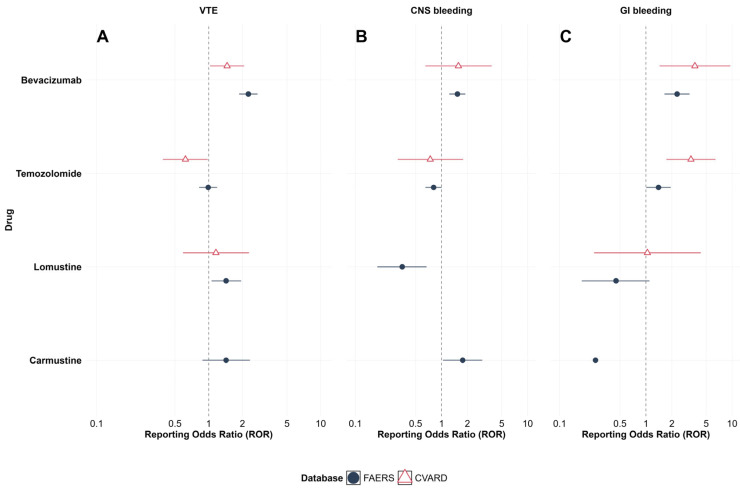
Forest plot of relative odds ratio (ROR) of venous thromboembolism and bleeding events related to four glioma treatment drugs (FAERS and CVARD). (**A**) Venous thromboembolism (VTE): RORs (with 95% CIs) for bevacizumab, temozolomide, lomustine, and carmustine; the vertical dashed line indicates the null (ROR = 1). (**B**) Central nervous system (CNS) bleeding: RORs (with 95% CIs) for the same four therapies, with the null reference line at ROR = 1. (**C**) Gastrointestinal (GI) bleeding: RORs (with 95% CIs) for the same four therapies, with the null reference line at ROR = 1.

To visualize these patterns, we plotted ROR-based forest plots for the three endpoints (Figure 2). In Figure 2A, bevacizumab shows RORs for VTE that are clearly >1 in both the FAERS and the CVARD, with 95% CIs not crossing the null line, highlighting a consistent VTE signal across databases. The RORs for temozolomide cluster around or below 1, and nitrosourea agents show only limited increase in selected settings, reinforcing the conclusion that bevacizumab has the most prominent thrombotic safety signal. In Figure 2B, bevacizumab demonstrates RORs for CNS bleeding that are consistently >1 in the FAERS, whereas the estimates in the CVARD are directionally similar but more imprecise, underscoring limited cross-database consistency for this endpoint; the ROR for carmustine in the FAERS is also slightly elevated but imprecise. Figure 2C further emphasizes the contrast in GI bleeding: bevacizumab has moderately to markedly increased RORs with 95% CIs entirely above 1 in both databases, while RORs for temozolomide and lomustine generally approximate 1 or are unstable. These visualizations support the conclusion that in real-world glioma treatment, bevacizumab is associated with the most pronounced VTE and GI bleeding signals, whereas the CNS bleeding signal is weaker and less consistent across databases.

### 3.3. Subgroup Analysis of Bevacizumab-Based Regimens

In the comparisons of bevacizumab-based regimens, the combination strategies generally showed stronger disproportionality signals for VTE and bleeding outcomes than bevacizumab monotherapy in both the FAERS and the CVARD (Table 4a–c). For VTE, the RORs for bevacizumab + temozolomide and bevacizumab + lomustine were 2.08 (95% CI: 1.66–2.60) and 2.37 (95% CI: 1.71–3.28), respectively, in the FAERS, and 1.83 (95% CI: 1.12–2.99) and 1.55 (95% CI: 1.01–2.40), respectively, in the CVARD, indicating that VTE reporting with bevacizumab-containing combination chemotherapy was approximately 1.5–2-fold higher than with bevacizumab monotherapy in both databases.

For CNS bleeding, the bevacizumab + temozolomide regimen had an ROR of 1.93 (95% CI: 1.47–2.54) relative to bevacizumab monotherapy in the FAERS, and an even higher ROR of 13.66 (95% CI: 5.71–32.67) in the CVARD. By contrast, RORs for bevacizumab + lomustine in both databases were close to 1, with wide CIs crossing 1, indicating no robust signal. A similar pattern was observed for GI bleeding: bevacizumab + temozolomide was associated with RORs of 2.53 (95% CI: 1.81–3.54) in the FAERS and 4.53 (95% CI: 2.36–8.70) in the CVARD, both clearly exceeding those for bevacizumab monotherapy, whereas bevacizumab + lomustine had RORs near 1 with 95% CIs spanning unity. Overall, among bevacizumab-based regimens, bevacizumab + temozolomide showed the most pronounced and cross-database-consistent excess reporting of VTE and GI bleeding, whereas bevacizumab + lomustine appeared to increase the VTE signal to a lesser extent and did not show a clear additional impact on bleeding outcomes. These findings suggest that intensified bevacizumab-containing regimens, particularly those combined with temozolomide, may entail a cumulative thrombotic and bleeding burden in glioma patients and warrant special attention in future research and clinical practice.

### 3.4. Sensitivity Analysis

In the restricted cohort including only reports with higher-certainty malignant glioma or glioblastoma diagnoses, bevacizumab-related thrombotic and bleeding disproportionality patterns were broadly similar to those observed in the main analysis (Table 5). For VTE, the number of bevacizumab-related VTE reports in the FAERS decreased from 284 to 256, and the ROR declined modestly from 2.26 (95% CI: 1.87–2.73) to 1.85 (95% CI: 1.51–2.26), but the lower bound of the 95% CI remained clearly >1. In the CVARD, the corresponding ROR increased from 1.46 (95% CI: 1.03–2.07) to 1.70 (95% CI: 1.05–2.77), and VTE signals remained positive in both databases under the stricter definition.

For CNS bleeding, the ROR for bevacizumab in the FAERS was almost identical between the main and restricted cohorts—1.53 (95% CI, 1.23–1.89) versus 1.53 (95% CI, 1.18–1.98)—consistently meeting the criteria for a positive signal. In the CVARD, however, RORs were imprecise in both analyses (1.57, 95% CI, 0.65–3.82 in the main analysis and 0.93, 95% CI, 0.48–1.83 in the restricted cohort), with wide CIs crossing 1, indicating no stable signal. For GI bleeding, the ROR for bevacizumab in the FAERS decreased modestly from 2.30 (95% CI, 1.64–3.21) to 1.63 (95% CI, 1.05–2.53) in the restricted cohort, and in the CVARD, it decreased from 3.70 (95% CI, 1.44–9.53) to 3.01 (95% CI, 1.01–9.00). In both databases, however, the lower bounds of the 95% CIs remained >1 in both the main and restricted analyses. Overall, using the predefined criterion of a ≥ 3 and an ROR 95% CI lower bound >1, bevacizumab-related VTE and GI bleeding signals remained present in both databases after narrowing the glioma case definition, whereas the CNS bleeding signal in the CVARD remained unstable due to small numbers. These findings support the robustness of the main results to reasonable variations in case definition.

In the bevacizumab-specific PS-only sensitivity analysis in the FAERS, restricting exposure to primary suspect reports reduced the number of bevacizumab-related ICSRs but did not materially change the direction or magnitude of disproportionality. For VTE, the PS-only ROR was 1.98 (95% CI 1.65–2.38) compared with 2.26 (95% CI 1.87–2.73) in the main analysis; for CNS bleeding, the PS-only ROR was 1.48 (95% CI 1.18–1.84) versus 1.53 (95% CI 1.23–1.89); and for GI bleeding, the PS-only ROR was 2.08 (95% CI 1.49–2.91) versus 2.30 (95% CI 1.64–3.21). In the CVARD, the PS-only analysis yielded elevated RORs for VTE (1.86, 95% CI 1.28–2.69 vs. 1.46, 95% CI 1.03–2.07 in the main analysis), while estimates for CNS and GI bleeding became less precise, with wider CIs including 1.0, reflecting limited case counts (Supplementary Table S1). Overall, these findings indicate that the positive disproportionality signals for bevacizumab-related VTE and GI bleeding are broadly robust to both case-definition and exposure-definition assumptions.

## 4. Discussion

Using the FAERS and the CVARD, we systematically evaluated disproportionality patterns linking four systemic therapies for glioma—temozolomide, bevacizumab, lomustine and carmustine—with three serious outcomes, i.e., VTE, CNS bleeding and GI bleeding, and further compared the safety profiles of bevacizumab monotherapy versus bevacizumab-based combination regimens. Our principal findings can be summarized as follows: First, among the four drugs studied, bevacizumab was the only agent that showed robust, moderate-to-strong positive signals for VTE and GI bleeding in both databases, whereas temozolomide generally displayed a neutral or slightly protective pattern for VTE, and nitrosoureas exhibited only limited, unstable signal increases in selected analyses. Second, bevacizumab was associated with a clear CNS bleeding signal in the FAERS, but database-level consistency for this endpoint was limited, as estimates in the CVARD were imprecise. Third, among bevacizumab-based treatments, the bevacizumab + temozolomide regimen showed substantially stronger signals for VTE and GI bleeding than bevacizumab monotherapy, while bevacizumab + lomustine appeared to confer only a modestly higher VTE signal and no clear additional bleeding signal. These patterns persisted in sensitivity analyses using a stricter malignant glioma/glioblastoma case definition, supporting the robustness of our main conclusions.

Our findings are broadly consistent with and extend existing evidence indicating increased thrombotic and bleeding signals with anti-VEGF therapies. Multiple randomized trials and meta-analyses in solid tumors have reported elevated rates of arterial and venous thromboembolic events, GI bleeding and even GI perforation associated with bevacizumab [21,22]. Furthermore, a real-world nested case–control study specifically in high-grade glioma also identified an increased risk of VTE associated with bevacizumab use [23]. However, data specifically addressing glioma populations are relatively scarce and mainly come from clinical trials with restrictive eligibility criteria and limited real-world generalizability. By leveraging spontaneous reporting data from two large pharmacovigilance systems and focusing on a glioma cohort, we concurrently examined temozolomide, traditional nitrosoureas and bevacizumab. We found that bevacizumab showed stronger disproportionality signals for VTE and GI bleeding than other therapies, with reporting odds ratios (RORs) approximately 1.5–3 times higher and consistent directions across both the FAERS and the CVARD, thereby refining the relative positioning of the thrombotic and bleeding risks of bevacizumab in the glioma setting from a pharmacovigilance perspective.

From a mechanistic standpoint, glioma is intrinsically associated with a highly prothrombotic state [24]. Tumor-derived procoagulant factors, tumor-related inflammation, neurosurgical procedures, and corticosteroid therapy jointly drive an elevated risk of VTE, while intracranial tumor infiltration and treatment-related thrombocytopenia predispose patients to bleeding [25,26]. In this high-risk context, bevacizumab may further disrupt the hemostatic balance by inhibiting VEGF-A signaling. Mechanistically, this inhibition can impair endothelial repair, reduce vasodilatory mediators such as nitric oxide and prostacyclin and promote vasoconstriction and platelet activation, thereby exacerbating vascular fragility and predisposing patients to both thrombotic and bleeding events [17]. These cross-database-consistent signals align with pharmacologic expectations and, given that bevacizumab is almost exclusively administered as standard-dose intravenous infusions in glioma practice, should be interpreted as reflecting risks under routine dosing rather than at specific plasma concentration thresholds [27].

In contrast, temozolomide did not exhibit a clear VTE signal and, in some analyses, had RORs below 1, suggesting that standard temozolomide chemoradiotherapy may not substantially amplify VTE risk beyond the already high baseline risk associated with glioma itself. The stronger GI bleeding signal for temozolomide observed in the CVARD compared with the FAERS likely reflects a combination of database- and system-level differences. The FAERS and the CVARD differ in reporting procedures, regulatory triggers and coding practices; oncology centers contributing to the CVARD may have a lower threshold or stronger incentives to report serious GI bleeding events, resulting in higher apparent disproportionality. In addition, we cannot systematically account for variation in concomitant medications—such as anticoagulants, antiplatelet agents and non-steroidal anti-inflammatory drugs—or in supportive care, including the routine use of proton pump inhibitors and other gastroprotective strategies, all of which may mitigate or modify GI bleeding propensity in temozolomide-treated patients. Demographic and clinical structures (e.g., age distribution, comorbidity burden and dosing patterns) may also differ among health care systems. Taken together, these factors underscore that cross-database differences in temozolomide-related GI bleeding disproportionality should be interpreted cautiously and considered hypothesis-generating only; further, they should not be overinterpreted in the absence of confirmatory cohort studies with detailed clinical covariates. Finally, regarding lomustine and carmustine, the lack of cross-database reproducibility and wide confidence intervals suggest that current evidence is insufficient to draw firm conclusions about their thrombotic and bleeding disproportionality patterns or relative safety profile. For carmustine, this uncertainty is further compounded by the fact that, in modern glioma practice, it is frequently delivered as Gliadel wafers with limited systemic absorption, while FAERS and CVARD do not reliably distinguish wafer-based from systemic use; consequently, the few carmustine-related VTE and GI bleeding reports in our cohort should be regarded as exploratory pharmacovigilance signals rather than a definitive characterization of the adverse event profile of BCNU wafers, for which CNS local complications are more clinically salient.

From a conceptual standpoint, disease severity, treatment line and cumulative toxicity are likely to act in concert to shape the background propensity for VTE and bleeding in malignant glioma [28]. Patients with more aggressive or recurrent disease who are treated in later lines of therapy typically have longer exposure to neurosurgery, radiotherapy, high-dose corticosteroids, alkylating chemotherapy and, in some cases, anti-angiogenic agents, all of which can progressively promote hypercoagulability, endothelial injury, thrombocytopenia and mucosal or vascular fragility [5]. As bevacizumab-based combinations, particularly bevacizumab plus temozolomide, are often reserved for such heavily pretreated, high-risk patients, their stronger disproportionality signals in the FAERS and the CVARD may partly reflect this cumulative risk environment and patient selection, in addition to any potential regimen-specific pharmacologic effects. Our analysis of bevacizumab-based combination regimens has important implications for clinical practice. We found that compared with bevacizumab monotherapy, bevacizumab + temozolomide regimens had consistently higher RORs for VTE and GI bleeding in both databases, whereas bevacizumab + lomustine showed only moderate VTE disproportional reporting increase and no robust bleeding signal. It should be emphasized that spontaneous reporting data do not provide detailed information on treatment lines, disease stage, prior therapies or performance status. Bevacizumab + temozolomide regimens may be more frequently used in patients with more advanced disease or in intensified treatment settings [29]. Their higher thrombotic and bleeding reporting proportions likely reflect a combination of disease severity, cumulative treatment burden and drug effects. We, therefore, interpret these findings as indicating that intensified bevacizumab-based regimens, particularly those combined with temozolomide, show a higher overall level of disproportional reporting for VTE and GI bleeding in real-world glioma practice, rather than attributing risk solely to a single agent. Nonetheless, the consistent direction of higher RORs for combination versus monotherapy regimens within the same glioma cohort, database and endpoint definition suggests that bevacizumab-containing combination chemotherapy should be regarded as high-risk for thrombotic and bleeding outcomes and that the benefits of disease control should be carefully balanced against potential harms.

Methodologically, this study has several strengths. First, by using two large spontaneous reporting databases from different health care systems, we were able to examine the cross-region consistency of drug–event signals and reduce the likelihood to obtain findings driven solely by the characteristics of a single system. Second, we prespecified four systemic therapies that are the most representative in glioma treatment [30], ensuring that comparisons were grounded in a coherent disease and treatment context and avoiding the pitfalls of pooling heterogeneous antineoplastic agents with diverse indications. Third, we focused on three clinically salient severe outcomes—VTE, CNS bleeding and GI bleeding—and used ROR as the primary disproportionality metric, with PRR, IC/IC_025_ and EBGM/EB_05_ as secondary references, which allowed us to cross-validate signal directionality without imposing overly conservative thresholds. Fourth, sensitivity analyses restricting the cohort to clearly malignant glioma/glioblastoma showed that bevacizumab-related VTE and GI bleeding signals remained largely unchanged, reinforcing the robustness of our findings to variations in case definition.

Nonetheless, several limitations inherent to spontaneous reporting systems must be acknowledged. First, the FAERS and the CVARD are passive surveillance tools subject to under-reporting, delayed and selective reporting, duplicate entries and substantial missing data. We could not obtain denominators for exposed populations or person-time; thus, disproportionality measures such as the ROR capture patterns of disproportionate reporting rather than true incidence rates or absolute risks, and differences in data structure, reporting culture and regulatory context between the FAERS and the CVARD may have contributed to cross-database discrepancies such as the temozolomide–GI bleeding association. Second, we were unable to systematically adjust for key clinical confounders, including disease severity, history of neurosurgery and cranial radiotherapy, concomitant medications (e.g., anticoagulants, antiplatelet agents and non-steroidal anti-inflammatory drugs), supportive care (e.g., use of proton pump inhibitors) and baseline comorbidities, because these variables are incompletely and heterogeneously recorded; in particular, cranial radiotherapy could not be consistently ascertained. Our endpoints, therefore, reflect events occurring against a background of prior or concomitant radiotherapy, with residual confounding by radiation exposure likely persisting. Third, the overall number of nitrosourea-related VTE and bleeding events was relatively small, resulting in wide confidence intervals and rendering findings for lomustine and carmustine exploratory rather than definitive. Fourth, the glioma cohort was defined using English-language free-text diagnostic terms without the systematic capture of non-English terminology or uncommon abbreviations, so some misclassification (including the inclusion of lower-grade or non-malignant CNS tumors and the exclusion of some true malignant gliomas) is inevitable and would be primarily expected to dilute the signals rather than create disproportionality. Fifth, exposure to each study drug was defined by any mention in the ICSR (suspect or concomitant), and information on whether VTE or bleeding episodes were incident or recurrent was not available; as a result, our endpoints combine first and recurrent, often more complicated events, and serious outcomes are likely over-represented among reported cases, particularly for temozolomide. Taken together, these constraints mean that our analyses cannot provide radiotherapy-adjusted or incidence-based risk estimates and should instead be interpreted strictly as hypothesis-generating pharmacovigilance signals of disproportionate reporting that require confirmation in well-designed observational or interventional studies.

## 5. Conclusions

In summary, using dual-database spontaneous reporting data, we delineated the VTE and bleeding disproportionality profiles of four commonly used systemic therapies in glioma. Bevacizumab emerged as the drug with the most prominent real-world disproportionality signals for VTE and GI bleeding, whereas temozolomide did not show an additional VTE signal beyond the already high baseline risk associated with glioma. Notably, bevacizumab-containing combination regimens—particularly those combined with temozolomide—showed stronger VTE and GI bleeding signals than bevacizumab monotherapy. While these patterns suggest that closer vascular monitoring may be warranted for such regimens, we acknowledge that these stronger signals likely reflect, at least in part, confounding by indication, as patients receiving combination therapies typically have more aggressive disease and heavier cumulative treatment burdens. Future studies combining prospective cohorts and mathematical modeling strategies [31], with careful control for disease severity, are needed to further quantify the incidence and prognostic impact of these events and guide the balance between tumor control and vascular risks in this high-risk population.

## Figures and Tables

**Table 1:** Baseline characteristics of glioma treatment-related reports in FAERS and CVARD.

Characteristic	FAERS Bevacizumab	FAERS Temozolomide	FAERS Lomustine	FAERS Carmustine	CVARD Bevacizumab	CVARD Temozolomide	CVARD Lomustine
Sex n (%)							
Male	183 (39.1%)	122 (35.7%)	23 (48.9%)	8 (29.6%)	77 (56.2%)	26 (70.3%)	5 (45.5%)
Female	100 (21.3%)	113 (33%)	7 (14.9%)	6 (22.2%)	39 (28.5%)	11 (29.7%)	4 (36.4%)
Unknown	185 (39.6%)	107 (31.3%)	17 (36.2%)	13 (48.2%)	21 (15.3%)		2 (18.1%)
Age							
12–17 years	5 (1%)	1 (0.2%)					
18–64 years	181 (38.6%)	132 (38.6%)	25 (53.2%)	7 (25.9%)	102 (74.4%)	28 (75.7%)	9 (81.8%)
65–85 years	71 (15.1%)	93 (27.2%)	5 (10.6%)	6 (22.2%)	10 (7.3%)	3 (8.1%)	
Unknown	211 (45.3%)	116 (34%)	17 (36.2%)	14 (51.9%)	25 (18.3%)	6 (16.2%)	2 (18.2%)
Weight							
<50 kg	10 (2.1%)	12 (3.5%)		1 (3.7%)			
50–100 kg	112 (23.9%)	96 (28.1%)	12 (25.5%)	7 (25.9%)	54 (39.4%)	15 (40.5%)	7 (63.6%)
>100 kg	14 (2.9%)	3 (0.8%)	4 (8.5%)	2 (7.4%)	9 (6.6%)		1 (9.1%)
Unknown	332 (71.1%)	231 (67.6%)	31 (66%)	17 (63%)	74 (54%)	22 (59.5%)	3 (27.3%)
Reports from health-care professionals, n (%)	302 (64.5%)	216 (63.1%)	24 (51.1%)	13 (48.1%)	74 (54%)	21 (56.7%)	8 (72.7%)
Serious cases, n (%)	82 (17.5%)	63 (18.4%)	11 (23.4%)	2 (7.4%)	21 (15.3)	9 (24.3%)	2 (18.2%)

**Table 2:** Disproportionality analysis for venous thromboembolism (VTE) (primary signal defined by ROR).

Database	Drug	a (Cases)	ROR (95% CI)	Signal by ROR	PRR (Chi-Square Value)	IC (IC_025_)	EBGM (EB_05_)
FAERS	Bevacizumab	284	2.26 (1.87–2.73)	Yes	2.23 (76.45)	0.57 (0.34)	1.48 (1.23)
FAERS	Temozolomide	202	0.99 (0.82–1.19)	No	0.99 (0.02)	−0.01 (−0.26)	0.99 (0.83)
FAERS	Lomustine	47	1.43 (1.06–1.95)	Yes	1.43 (5.47)	0.47 (0.01)	1.38 (1.02)
FAERS	Carmustine	17	1.43 (0.88–2.33)	No	1.42 (2.09)	0.49 (−0.24)	1.41 (0.86)
CVARD	Bevacizumab	110	1.46 (1.03–2.07)	Yes	1.45 (4.57)	0.18 (−0.31)	1.13 (0.79)
CVARD	Temozolomide	21	0.62 (0.39–0.98)	No	0.62 (4.18)	−0.56 (−1.21)	0.68 (0.43)
CVARD	Lomustine	9	1.16 (0.59–2.29)	No	1.16 (0.19)	0.2 (−0.78)	1.15 (0.58)

**Table 3:** (a) Disproportionality analysis for intracranial and gastrointestinal bleeding in FAERS (primary signal defined by ROR). (b) Disproportionality analysis for intracranial and gastrointestinal bleeding in CVARD (primary signal defined by ROR).

(a)								
Database	Drug	Event	a (Cases)	ROR (95% CI)	Signal by ROR	PRR (Chi-Square Value)	IC (IC_025_)	EBGM (EB_05_)
FAERS	Bevacizumab	CNS bleeding	174	1.53 (1.23–1.89)	Yes	1.52 (15.14)	0.32 (0.05)	1.25 (1.01)
FAERS	Bevacizumab	GI bleeding	90	2.3 (1.64–3.21)	Yes	2.29 (25.17)	0.58 (0.18)	1.49 (1.07)
FAERS	Temozolomide	CNS bleeding	130	0.81 (0.65–1)	No	0.81 (3.69)	−0.18 (−0.48)	0.88 (0.71)
FAERS	Temozolomide	GI bleeding	76	1.4 (1.01–1.94)	No	1.4 (4.11)	0.25 (−0.17)	1.19 (0.86)
FAERS	Lomustine	CNS bleeding	9	0.35 (0.18–0.67)	No	0.35 (10.79)	−1.45 (−2.29)	0.37 (0.19)
FAERS	Lomustine	GI bleeding	5	0.45 (0.18–1.1)	No	0.45 (3.26)	−1.09 (−2.17)	0.47 (0.19)
FAERS	Carmustine	CNS bleeding	15	1.76 (1.04–2.97)	Yes	1.75 (4.63)	0.78 (−0.03)	1.71 (1.02)
FAERS	Carmustine	GI bleeding	1	0.26 (0.04–1.85)	No	0.26 (2.12)	−1.92 (−3.33)	0.26 (0.04)
**(b)**								
**Database**	**Drug**	**Event**	**a (Cases)**	**ROR (95% CI)**	**Signal by ROR**	**PRR (Chi-Square Value)**	**IC (IC_025_)**	**EBGM (EB_05_)**
CVARD	Bevacizumab	CNS bleeding	26	1.57 (0.65–3.82)	No	1.57 (1)	0.15 (−0.65)	1.11 (0.45)
CVARD	Bevacizumab	GI bleeding	31	3.7 (1.44–9.53)	Yes	3.69 (8.47)	0.46 (−0.29)	1.37 (0.53)
CVARD	Temozolomide	CNS bleeding	6	0.74 (0.31–1.78)	No	0.74 (0.46)	−0.35 (−1.53)	0.78 (0.33)
CVARD	Temozolomide	GI bleeding	17	3.33 (1.73–6.42)	Yes	3.31 (14.55)	1.15 (0.19)	2.22 (1.15)
CVARD	Lomustine	GI bleeding	2	1.04 (0.25–4.33)	No	1.04 (0)	0.05 (−1.73)	1.04 (0.25)

ROR served as the primary measure of disproportionality. Signal detected by ROR: A signal was defined as “Yes” if the count (a) was ≥3 and the lower limit of the 95% confidence interval for the ROR was >1; otherwise, it was defined as “No”. Counts represent the number of ICSRs with ≥1 VTE (or CNS bleeding) PT and not the number of clinical events. Abbreviations: CNS, central nervous system; GI, gastrointestinal.

**Table 4:** (a) Comparison of bevacizumab-based regimens for venous thromboembolism in FAERS and CVARD. (b) Comparison of bevacizumab-based regimens for CNS bleeding in FAERS and CVARD. (c) Comparison of bevacizumab-based regimens for GI bleeding in FAERS and CVARD.

(a)				
Database	Regimen	Event	a (Cases)	ROR vs. BEV Mono (95%CI)
FAERS	Bevacizumab mono	VTE	179	Reference
FAERS	Bevacizumab + Temozolomide	VTE	105	2.08 (1.66–2.60)
FAERS	Bevacizumab + Lomustine	VTE	42	2.37 (1.71–3.28)
CVARD	Bevacizumab mono	VTE	85	Reference
CVARD	Bevacizumab + Temozolomide	VTE	22	1.83 (1.12–2.99)
CVARD	Bevacizumab + Lomustine	VTE	32	1.55 (1.01–2.40)
**(b)**				
**Database**	**Regimen**	**Event**	**a (Cases)**	**ROR vs. BEV Mono (95%CI)**
FAERS	Bevacizumab mono	CNS bleeding	108	Reference
FAERS	Bevacizumab + Temozolomide	CNS bleeding	66	1.93 (1.47–2.54)
FAERS	Bevacizumab + Lomustine	CNS bleeding	8	1.14 (0.56–2.32)
CVARD	Bevacizumab mono	CNS bleeding	22	Reference
CVARD	Bevacizumab + Temozolomide	CNS bleeding	8	13.66 (5.71–32.67)
CVARD	Bevacizumab + Lomustine	CNS bleeding	1	0.99 (0.13–7.53)
**(c)**				
**Database**	**Regimen**	**Event**	**a (Cases)**	**ROR vs. BEV Mono (95%CI)**
FAERS	Bevacizumab mono	GI bleeding	36	Reference
FAERS	Bevacizumab + Temozolomide	GI bleeding	54	2.53 (1.81–3.54)
FAERS	Bevacizumab + Lomustine	GI bleeding	5	1.43 (0.58–3.52)
CVARD	Bevacizumab mono	GI bleeding	15	Reference
CVARD	Bevacizumab + Temozolomide	GI bleeding	16	4.53 (2.36–8.70)
CVARD	Bevacizumab + Lomustine	GI bleeding	4	0.99 (0.34–2.89)

Abbreviations: CNS, central nervous system; GI, gastrointestinal.

**Table 5:** Sensitivity analysis of bevacizumab-related thrombotic and bleeding events in the main vs. restricted glioma cohort (FAERS and CVARD).

Event	Database	N_main	ROR_main (95% CI)	Signal_main	N_strict	ROR_strict (95% CI)	Signal_strict
VTE	FAERS	284	2.26 (1.87–2.73)	Yes	256	1.85 (1.51–2.26)	Yes
VTE	CVARD	110	1.46 (1.03–2.07)	Yes	94	1.70 (1.05–2.77)	Yes
CNS bleeding	FAERS	174	1.53 (1.23–1.89)	Yes	142	1.53 (1.18–1.98)	Yes
CNS bleeding	CVARD	26	1.57 (0.65–3.82)	No	22	0.93 (0.48–1.83)	No
GI bleeding	FAERS	90	2.3 (1.64–3.21)	Yes	50	1.63 (1.05–2.53)	Yes
GI bleeding	CVARD	31	3.7 (1.44–9.53)	Yes	25	3.01 (1.01–9.00)	Yes

Abbreviations: VTE, venous thromboembolism; CNS, central nervous system; GI, gastrointestinal. Signal_main/Signal_strict: defined as a ≥ 3 and lower bound of 95% CI for ROR > 1.

## Data Availability

The data underlying this article will be shared on reasonable request to the corresponding author.

## Supplementary Materials

The following supporting information can be downloaded at: https://www.mdpi.com/article/10.3390/cancers18010130/s1, Table S1: Sensitivity analysis restricting bevacizumab exposure to primary suspect reports for VTE, CNS bleeding and GI bleeding in FAERS and CVARD.
