# Supplementary material for: Venous Thromboembolism and Bleeding with Temozolomide-, Bevacizumab-, and Nitrosourea-Based Therapy in Glioma: A Dual-Database Pharmacovigilance Study
Source: Cancers (Basel). 2025 Dec 30;18(1):130. doi: 10.3390/cancers18010130 (PMC12784670; doi:10.3390/cancers18010130)
Supplement: Supplementary file 1 [file cancers-18-00130-s001.zip › cancers-4050776-supplementary.pdf]

Supplementary Table S1. Sensitivity analysis restricting bevacizumab exposure to primary suspect reports for VTE, CNS bleeding and GI bleeding in FAERS and CVARD

Class	Drug	N_main	FAERS ROR_main (95% CI)	N_PS_only	FAERS PS_only ROR (95% CI)	N_main	CVARD ROR_main (95% CI)	N_PS_only	CVARD PS_only ROR (95% CI)
VTE	Bevacizumab	284	2.26 (1.87 - 2.73)	209	1.98 (1.65 - 2.38)	110	1.46 (1.03 - 2.07)	94	1.86 (1.28-2.69)
CNS bleeding	Bevacizumab	174	1.53 (1.23 - 1.89)	128	1.48 (1.18 - 1.84)	26	1.57 (0.65 - 3.82)	21	0.93 (0.48 - 1.83)
GI bleeding	Bevacizumab	90	2.3 (1.64 - 3.21)	80	2.08 (1.49-2.91)	31	3.7 (1.44 - 9.53)	22	2.63 (0.99- 6.94)

Abbreviations: FAERS, FDA Adverse Event Reporting System; CVARD, Canada Vigilance Adverse Reaction Database; VTE, venous thromboembolism; CNS, central nervous system; GI, gastrointestinal; ROR, reporting odds ratio; CI, confidence interval; ICSR, individual case safety report; PS, primary suspect.

N_main indicates the number of ICSRs with bevacizumab exposure in the main analysis, where exposure was defined as any mention of bevacizumab (primary suspect, secondary suspect or concomitant); ROR_main denotes the corresponding reporting odds ratio and 95% CI. N_PS_only and PS_only ROR represent the number of ICSRs and RORs when bevacizumab exposure was restricted to reports in which bevacizumab was coded as a primary suspect drug. For each endpoint, an ICSR was counted once if it contained ≥1 relevant MedDRA preferred term from the prespecified VTE, CNS bleeding or GI bleeding PT group. Estimates with wide CIs, particularly for bleeding endpoints in CVARD, should be interpreted with caution due to limited case counts.
